# Identification of Biallelic dystrophin gene variants during maternal carrier testing for Becker muscular dystrophy and review of the *DMD* exon 49–51 deletion phenotype

**DOI:** 10.1002/mgg3.2088

**Published:** 2022-11-24

**Authors:** Elizabeth A. Ulm, Chinmayee B. Nagaraj, Cuixia Tian, Teresa A. Smolarek

**Affiliations:** ^1^ Division of Human Genetics Cincinnati Children's Hospital Medical Center Cincinnati Ohio USA; ^2^ Division of Neurology Cincinnati Children's Hospital Medical Center Cincinnati Ohio USA; ^3^ Department of Pediatrics University of Cincinnati School of Medicine Cincinnati Ohio USA

**Keywords:** Duchenne muscular dystrophy, genetic diseases, neuromuscular diseases, X‐Linked dilated cardiomyopathy

## Abstract

**Background:**

Dystrophinopathies are X‐linked recessive conditions caused by pathogenic variants in the dystrophin (*DMD*) gene. In a family that included two boys with Becker muscular dystrophy (BMD) due to a *DMD* deletion of exons 45–47, maternal carrier testing unexpectedly identified biallelic *DMD* deletions of exons 45–47 and 49–51.

**Methods:**

The patient's mild phenotype in the setting of biallelic *DMD* variants prompted further investigation of the exon 49–51 deletion in particular, via literature review and retrospective chart review of patients who have been evaluated in our institution's comprehensive neuromuscular center and/or diagnosed in our clinical genetic testing laboratory.

**Results:**

To our knowledge, this is only the fifth case of confirmed biallelic *DMD* variants in a female. In males, the *DMD* exon 49–51 deletion appears to result in a mild BMD phenotype with low or normal creatine kinase levels. This deletion comprised 19% (4/21) of dystrophinopathies diagnosed by chromosomal microarray (CMA) in males during the past ten years in our clinical laboratory. Most individuals identified by chart review were diagnosed through CMA, despite the fact that microarray was genome‐wide and not *DMD*‐specific. This case raised important genetic counseling issues.

**Conclusion:**

The *DMD* exon 49–51 deletion appears to cause a variable but generally mild BMD phenotype. Its relatively frequent detection by CMA suggests it may be underdiagnosed.

## Graphical abstract

This manuscript summarizes a case of biallelic dystrophin (*DMD*) variants in a mildly affected female patient and additionally highlights the phenotypic spectrum of one of the patient's two *DMD* variants, a deletion of exons 49–51. This deletion previously had few reports in the published literature, despite its frequency within our institution's neuromuscular clinic. We identified 15 additional cases of this deletion through our retrospective chart review, adding considerable knowledge to the 27 cases we initially located through a literature search.

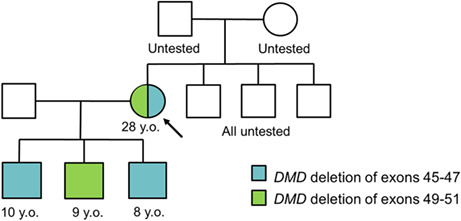

## 1 INTRODUCTION

Dystrophinopathies are allelic X‐linked recessive conditions caused by pathogenic variants in the dystrophin (*DMD*) gene (OMIM 300377). In males, a hemizygous *DMD* variant may result in Duchenne muscular dystrophy (DMD), Becker muscular dystrophy (BMD), or isolated X‐linked dilated cardiomyopathy (XLDCM). Females who carry a heterozygous *DMD* variant are at increased risk for cardiomyopathy and may have mild skeletal muscle symptoms (Muntoni et al., 2003).

The spectrum of *DMD* gene variants includes large deletions of one or more exons (~67%), large duplications of one or more exons (~11%), and small sequence changes such as nonsense, frameshift, missense, or splice site alterations (~22%) (Tuffery‐Giraud et al., 2009).

In males, the distinction between the DMD and BMD phenotypes is predictable in greater than 90% of cases based on the *DMD* variant's expected impact on the dystrophin protein's translational reading frame. Out‐of‐frame variants typically result in DMD, while in‐frame variants typically result in BMD (Tuffery‐Giraud et al., 2009). The worldwide prevalence of DMD is estimated to be 4.78 per 100,000 males, compared to 1.53 per 100,000 males for BMD (Mah et al., 2014).

The distinction between BMD and XLDCM is frequently accounted for by the presence of skeletal muscle disease in the former and cardiomyopathy without skeletal muscle disease in the latter. As skeletal muscle disease in BMD may onset as late as adulthood, differentiation between these two phenotypes in the pediatric setting is not well defined, as they fall on the same disease spectrum. In some cases, the same *DMD* variant may result in either a BMD or XLDCM phenotype, although some mutations have been shown to confer earlier or later DCM risk (Ferlini et al., 2013; Kaspar et al., 2009; Mavrogeni et al., 2018; Towbin et al., 1993).

The recommended testing strategy to confirm the diagnosis of a dystrophinopathy in a male patient typically begins with deletion/duplication analysis of the *DMD* gene, followed by *DMD* sequencing if negative (Birnkrant et al., 2018). Deletions and duplications may be identified through multiplex ligation‐dependent probe amplification or array comparative genomic hybridization (aCGH), and deletions also have been historically identified through multiplex PCR. Sequence changes may be identified through Sanger sequencing (Birnkrant et al., 2018). Next‐generation sequencing methodologies that are optimized to assess for copy number changes can now identify deletions/duplications and sequence changes in a single assay (Nallamilli et al., 2021).

While not specifically optimized for detecting exon‐level *DMD* deletions and duplications, chromosomal microarray (CMA) has the ability to detect many *DMD* copy number changes, even though it is a broad first‐tier test to assess for copy number changes responsible for developmental disabilities or congenital anomalies (Miller et al., 2010), and is not specifically indicated to evaluate for dystrophinopathies.

Biallelic *DMD* variants in females, which theoretically may result in similar phenotypes as those of affected males, have been confirmed in at least four previously published cases. Two females with a clinical diagnosis of Duchenne muscular dystrophy have been described with biallelic out‐of‐frame *DMD* variants, one with uniparental isodisomy of the X chromosome in conjunction with a homozygous deletion of exon 50 (Quan et al., 1997) and a second with compound heterozygous *DMD* deletions of exons 48–50 and exons 51–53 (Takeshita et al., 2017). A homozygous in‐frame deletion of exons 45–55 has been reported in a female with Becker muscular dystrophy (Fujii et al., 2009). An asymptomatic female has been reported with a *DMD* duplication of exons 1–34 on one allele and a *DMD* deletion of exons 16–29 on the other allele (Onore et al., 2021). The fifth and six cases of females with two *DMD* variants, but without described phase, have also been published, one with severe BMD and the other without symptoms (Nozoe et al., 2016; Soltanzadeh et al., 2010). Additionally, in our genetics laboratory, we have detected one female patient with toe walking, tight heel cords and prominent muscle bulk with two de novo *DMD* duplications of unknown phase (Table 1).

**TABLE 1:** Cases of females with multiple *DMD* copy number variations

Publication	Clinical information	Genotype	Phenotype
Quan et al. (1997)	6 yo with calf pain	Homozygous de novo deletion of exon 50 (out of frame) in the *DMD* gene due to uniparental isodisomy of the X chromosome	DMD
Fujii et al. (2009)	7 yo with exercise intolerance, recurrent myoglobinuria	Homozygous deletion of exons 45–55 (in‐frame) in the *DMD* gene. Both parents carried the deletion.	BMD
Takeshita et al. (2017)	21 yo with Gowers' sign, calf hypertrophy and elevated CK identified at two years. Lost ambulation at 11 years.	*DMD* deletion of exons 48–53 identified by multiplex ligation‐dependent probe amplification, which were further resolved and determined to be biallelic variants: deletion of exons 48–50 on one allele and exons 51–53 on the opposite allele.	DMD
Nozoe et al. (2016)	62 yo with adult‐onset lower extremity weakness; CK 779 U/L; son with DMD	Duplications of exons 42–44 and 48–52 (*phase not described*).	Not specified
Soltanzadeh et al. (2010)	15 yo with muscle weakness onset at age 3 years and cardiomyopathy	Heterozygous out‐of‐frame deletion of exons 8–13 and heterozygous splice site variant c.10086 + 2 T > C (*phase not described*) (NM_004006.2)	severe BMD
Onore et al. (2021)	32 yo without symptoms	*DMD* duplication of exons 1–34 on one allele and deletion of exons 16–29 on the other allele.	None
This study	4 yo with toe walking, tight heel cords and prominent muscle bulk	*DMD* duplication of exons 8–9 and duplication of exons 61–79 *(phase unknown; both variants* de novo*)*	Symptomatic carrier

Abbreviations: BMD, Becker muscular dystrophy; DMD, Duchenne muscular dystrophy.

## 2 CASE

**FIGURE 1:**
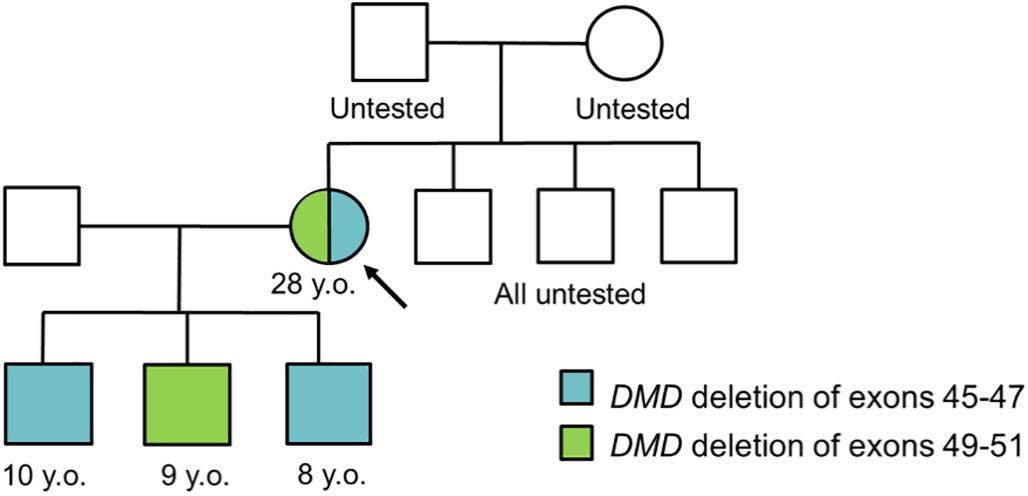
Pedigree

A 28‐year old mother of two boys who carry a diagnosis of Becker muscular dystrophy underwent routine maternal carrier testing via deletion/duplication analysis of the *DMD* gene by aCGH. Her oldest and youngest sons previously were diagnosed via aCGH at ages five and seven years with a hemizygous in‐frame *DMD* deletion of exons 45–47. The mother was confirmed to carry the exon 45–47 deletion in the heterozygous state, but a second deletion, of exons 49–51, was unexpectedly identified during testing. The mother reported muscle cramps and pain but no muscle weakness. Cardiac MRI for her showed no current evidence of cardiomyopathy. There was a reported family history of muscle cramps in her brothers, but no other family members had undergone *DMD* testing to date (Figure 1).

The two deletions were confirmed to be in *trans* upon the identification of the exon 49–51 deletion in her middle son by aCGH. He was six years old at the time of his brothers' diagnoses and previously had been reported to have normal creatine kinase (CK) levels by the family. He had also been perceived to be asymptomatic by the family compared to his brothers, and had not been evaluated clinically. Subsequent clinical evaluation at age nine years showed mildly elevated CK levels, with reported symptoms of muscle cramps and fatigue, consistent with Becker muscular dystrophy.

In light of its detection in the compound heterozygous state with a second deletion in a mildly affected female, we sought to better characterize the phenotype associated with the *DMD* exon 49–51 deletion via literature review and retrospective chart review. Unlike the exon 45–47 deletion, which is well‐described in the literature and is estimated to account for approximately 25% of in‐frame *DMD* deletions (Anwar et al., 2021) and between 2.2% and 28% of cases of BMD (Ling et al., 2020; Mah et al., 2011; Tuffery‐Giraud et al., 2009), the exon 49–51 deletion is described infrequently, despite its location in the hotspot region that involves exons 45–55 (Muntoni et al., 2003). Its detection in multiple patients by CMA in our genetics laboratory suggested it may be more frequent than accounted for by previous publications.

## 3 METHODS

### 3.1 Ethical compliance

The retrospective chart review was reviewed by the Cincinnati Children's Hospital Medical Center Institutional Review Board. A waiver of consent was granted and this study was determined to be exempt. We searched the PubMed database and Google Scholar using the keywords “DMD 49–51”, Duchenne muscular dystrophy 49–51,” “dystrophin 49–51″, and “Becker muscular dystrophy 49‐51”, and visited the DMD page from the Leiden muscular dystrophy pages (https://databases.lovd.nl/shared/genes/DMD) in order to collect published cases of the exon 49–51 deletion. Additionally, we performed a retrospective chart review of previous cases of the exon 49–51 deletion evaluated by our institution's comprehensive neuromuscular center and/or diagnosed by CMA by collecting the following metrics: indications for testing, age at diagnosis, test methodology, creatine kinase level ranges, physical exam findings, and family history.

## 4 RESULTS

We identified at least 27 instances of the *DMD* exon 49–51 deletion in males in the published literature that included phenotypic information (Chaudhary et al., 2009; Clemens et al., 2020; Guo et al., 2015; Kapoor et al., 2012; Ling et al., 2020; Mah et al., 2011; Mital et al., 1998; Muntoni et al., 1993; Muntoni et al., 1997; Rani et al., 2013; Tuffery‐Giraud et al., 2009; Waldrop et al., 2020). Table 2 shows published cases of the *DMD* exon 49–51 deletion identified during our literature review. Publications that listed this deletion without a phenotypic designation, including in the setting of prenatal testing, were excluded. Reported phenotypes in the remaining publications include: absence of symptoms (1), X‐linked DCM (3), BMD (14), intermediate MD (1), DMD (6), and “mild” MD (not categorized further) (1). When specified, creatine kinase levels were normal (in one instance of X‐linked DCM), or elevated (in the four remaining cases in which CK was described). We attempted to avoid duplicating cases that were represented in more than one publication.

**TABLE 2:** Cases of *DMD* exon 49–51 deletion identified during literature review which specified a phenotype characterization

Publication	Cases	Test methodology and reference sequence (if provided)	CK	Muscle biopsy	Clinical notes	Phenotype
Chaudhary et al. (2009)	1	Multiplex PCR	22,000 u/L	NS	Age at last evaluation: 5 yrs	“mild” NS
Clemens et al. (2020)	3	NS	NS	NS	NS	BMD (3)
Guo et al. (2015)	1	MLPA (NM_004006.2)	NS	NS	NS	DMD
Kapoor et al. (2012)	1	Multiplex PCR and MLPA	3218 u/L (ref <170 u/L)	Variation in fiber diameter; evidence of internal nuclei; occasional myophagocytosis; regenerating fiber and splitting. Absence of dystrophin staining on immunohistochemistry.	Separate additional clinical diagnosis of Waardenburg syndrome	DMD
Ling et al. (2020)	2	MLPA	NS	NS	Ages 4 and 5 yrs at diagnosis	BMD (2)
Mah et al. (2011)	2	MLPA or multiplex PCR (NS)	NS	NS	NS	DMD, MD
Mital et al. (1998)	1	Multiplex PCR	>1000 u/L	NS	Age of onset: 2–3 yrs; age at diagnosis: 7 yrs; loss of ambulation: 11 yrs	DMD
Muntoni et al. (1993)	1	Multiplex PCR and Southern blotting	NS	Pattern of fiber labeling: “94% continuous, variable intensity; 6% discontinuous, moderate intensity (*reduction in immunostaining was detected using C‐terminal antibodies or antibodies distal to a deleted portion of the protein)”	NS	BMD
Muntoni et al. (1997)	1	cDNA PCR	84 u/L	Cardiac biopsy: fibrosis, fiber size variability, strong immunostaining for N terminal, mid‐rod, and C terminal dystrophin domains. No staining using terminal rod domain antibodies. Western blot analysis: slight reduction in dystrophin expression, small molecular weight decrease.	Cardiac failure diagnosed at 50 yrs; normal skeletal muscle strength	XLDCM
Rani et al. (2013)	1	MLPA	4363 u/L	NS	Age of onset: 6 yrs; at last evaluation: 7 yrs with calf pseudohypertrophy	BMD
Tuffery‐Giraud et al. (2009)	1	NS	NS	dystrophin 1 and 2 – Reduced size, high quantity	Age of onset: 41 yrs	BMD
	1	NS	NS	NS	Age of onset: 1 yr	BMD
Waldrop et al. (2020)	11	Combination of multiplex PCR, aCGH, MLPA; two diagnosed incidentally by CMA	Elevated in 3, NS in 9	Described in 2: “reduced size, high quantity” and “rare revertant fibers”	NS	Asymptomatic (1) DMD (2) Intermediate MD (1) BMD (5) XLDCM (2)

Abbreviations: aCGH, array comparative genomic hybridization; BMD, Becker muscular dystrophy; cDNA PCR, complementary DNA polymerase chain reaction; CMA, chromosomal microarray (genome‐wide and not specific to *DMD*); DMD, Duchenne muscular dystrophy; Intermediate MD, intermediate muscular dystrophy; MD, unspecified phenotype; MLPA, multiplex ligation‐dependent probe amplification; Multiplex PCR, multiplex polymerase chain reaction; NS, not specified; u/L, units/liter; XLDCM, X‐linked dilated cardiomyopathy.

In our retrospective chart review, we identified 15 males with the *DMD* exon 49–51 deletion who were either evaluated in our comprehensive neuromuscular center and/or ascertained via CMA in our laboratory of genetics and genomics. Table 3 summarizes clinical features of these patients and their ages at last evaluation. Of male patients in our institutional cohort, muscle symptoms ranged from exercise myalgias, fatigue, and muscle tightness to absence of symptoms at most recent clinical evaluation. Cardiac evaluations were normal in 11 patients. Of the remaining four, one had an atrioventricular septal defect and atrioventricular canal defect attributable to his concurrent trisomy 21 diagnosis. Findings in the other three patients included mild right ventricular systolic dysfunction with mild aortic root dilation, mild left ventricular dilation, and focal myocardial fibrosis with borderline right and left ventricular systolic function. Ages at last clinical evaluation ranged from 3–15 years. Muscle weakness was not noted in any male patients, although it was submitted as the testing indication for one female patient in whom this deletion was identified in our laboratory by CMA (Table 4).

**TABLE 3:** Cases of *DMD* exon 49–51 deletion identified during retrospective chart review

Family	Test methodology	CK (lowest and highest)	Muscle biopsy	Muscle MRI	Clinical notes and genetic testing	Cardiac screening	Age at last evaluation	Phenotype
1	CMA	NS	None	None	Fragile X testing (normal) and CMA ordered during autism evaluation at 6 yrs; not clinically evaluated for dystrophinopathy subsequent to CMA result.	None	None	Unknown
2	NGS	249 (22–210 unit/L) 275 (22–210 unit/L)	None	None	Diagnosed at age 9 yrs after carrier testing in mother identified two variants in trans; intermittent calf pain noted at 8 years.	Normal echocardiogram, normal cardiac MRI	9 yrs	BMD
3	CMA then NGS	202 (22–210 unit/L) 171 (22–210 unit/L)	None	None	Fragile X testing (normal) and CMA ordered during evaluation for developmental delays at age 4 yrs; muscle pain and fatigue noted upon clinical evaluation.	Normal echocardiogram	6 yrs	BMD
4	CMA	227 (50–350 unit/L) 186 (32–189 unit/L)	None	None	Fragile X testing (normal) and CMA ordered for ADHD and speech/language delay; family history of muscle weakness among maternal family members (untested); stable motor function at age 15.	Normal echocardiogram, normal cardiac MRI	15 yrs	BMD
5	aCGH	62 (50–270 unit/L) 37 (60–305 unit/L)	None	None	Family history of BMD in brother and mother is a known carrier. Concurrent diagnosis of trisomy 21 with significant developmental delays. *DMD* gene testing ordered at birth due to his known family history.	Atrioventricular septal defect (AVSD)/atrioventricular canal defect (AVC) (concurrent diagnosis of trisomy 21)	3 yrs	BMD
	CMA then aCGH	749 (30–250 unit/L) 295 (32–189 unit/L)	20–100% of muscle dystrophin of decreased molecular size	None	Chromosome analysis (46, XY), Fragile X testing (normal), and CMA ordered for congenital heart defect, feeding difficulties. Clinical exam with stable motor function and muscle strength, some increased muscle tightness.	Mild right ventricular systolic dysfunction, mild aortic root dilation	15 yrs	BMD
6	aCGH	180 (30–250 unit/L) 686 (30–250 unit/L)	None	None	Diagnosed based on family history of carrier status in sister, identified by CMA ordered for developmental delays. Normal motor strength and function on exam.	Normal echocardiogram	6 yrs	BMD
7	CMA then aCGH	391 (30–250 unit/L) 1192 (32–189 unit/L)	None	No evidence of fatty infiltration or muscle edema/inflammatory changes within the imaged muscles of the pelvis / lower extremities.	CMA ordered based on clinical diagnosis of Asperger's syndrome. Normal motor function at age 14 yrs, significant muscle cramps and stiffness noted in the mornings; possible family history of BMD in maternal family members (untested).	Mild left ventricular dilation with normal systolic function (cardiac MRI)	15 yrs	BMD
8	CMA, aCGH	374 (75–215 unit/L) 161 (30–250 unit/L)	None	None	Chromosome analysis (46, XY), Fragile X testing (normal), and CMA ordered at the age of 4 yrs for developmental delay. Has hypotonia, exercise intolerance, exercise myalgias and normal motor function noted at 7 yrs.	Normal echocardiogram	7 yrs	BMD
9	CMA, NGS	1477 (22–210 unit/L)	None	None	CMA ordered during autism evaluation at 4 yrs; no gross motor delay or weakness.	Normal echocardiogram	4 yrs	BMD
	NGS	262 (32–189 unit/L)	None	None	Diagnosed after brother was identified with this deletion as an incidental finding. Asymptomatic aside from bilateral leg pain in the setting of heavy exercise.	Normal cardiac MRI	13 yrs	BMD
10	CMA, aCGH	141 (30–250 unit/L) 256 (30–250 unit/L)	None	None	CMA ordered for microcephaly, short stature, failure to thrive. Clinical evaluation: mild generalized hypotonia, bilateral pes planus, toe walking, normal muscle bulk, no weakness.	Normal echocardiogram	7 yrs	BMD
11	CMA, NGS	268 (32–189 unit/L) 420 (32–189 unit/L)	None	None	CMA ordered for developmental regression and hypotonia; has since been diagnosed with autism and has undergone non‐diagnostic mitochondrial DNA testing and a non‐diagnostic multi‐gene autism/intellectual disability/developmental delay panel. Normal neuromuscular exam with normal muscle strength and stable motor function and timed tests, but with muscle cramps and poor endurance.	Cardiac MRI with subclinical evidence of cardiomyopathy with focal myocardial fibrosis and borderline right and left ventricular systolic function	16 yrs	BMD
12	Not available	583 (75–215 unit/L) 354 (75–215 unit/L)	None	No muscle signal abnormality within the pelvis or proximal thighs to suggest inflammation or fatty infiltration.	Diagnosed at age 4 yrs following diagnosis of older brother. Normal motor strength and function.	Normal cardiac MRI	7 yrs	BMD
	Not available	947 (75–215 unit/L) 354 (75–215 unit/L)	None	No muscle signal abnormality within the pelvis or proximal thighs to suggest inflammation or fatty infiltration.	*DMD* deletion/duplication analysis ordered after elevated CK levels were observed following a febrile illness at age 7 years. Neuromuscular exam demonstrates tight hamstrings, exercise intolerance, muscle cramps.	Normal cardiac MRI	11 yrs	BMD

Abbreviations: aCGH, array comparative genomic hybridization; BMD, Becker muscular dystrophy; CMA, chromosomal microarray (genome‐wide and not specific to *DMD*); NGS, next‐generation sequencing; NS, not specified; u/L, units/liter.

**TABLE 4:** *DMD* copy number variants identified by CMA in the CCHMC genetics and genomics diagnostic laboratory between 1/1/2010–12/31/2020

Sex	Genomic coordinates (GRCh37/hg19)	Indication(s) for testing	Type of CNV	Exon(s) involved
**M**	chrX:31088893–31241938	Autism spectrum disorder, developmental delay, mixed receptive‐expressive language disorder, sensory dysfunction	Duplication	64–79
**M**	chrX:31310822–31647841	Advanced maternal age	Duplication	55–62
**M**	chrX:31900869–31996334	Becker muscular dystrophy, Dandy Walker malformation, facial dysmorphism, developmental delay, focal segmental glomerulosclerosis, sensorineural hearing loss	Deletion	45–47
**M**	chrX:32219959–32612294	Sensory dysfunction, motor skills disorder, hyperactive	Duplication	14–44
**M**	chrX:31868633–31919659	Leg length discrepancy, extremity deformity, hemihypertrophy	Deletion	48
**M**	chrX:33113049–33301223	Motor developmental delay, language disorder, encephalopathy	Duplication	1
**M**	chrX:31811505–31852903	Mixed receptive‐expressive language disorder, microcephaly, failure to thrive, developmental delay	Deletion	50
**M**	chrX:32616532–33732702	Developmental delay	Duplication	1–12
**F**	chrX:31696892–31969744	Ventricular septal defect	Duplication	46–53
**M**	chrX:31533207–31983162	Gross motor developmental delay	Deletion	46–55
**M**	chrX:32919897–33501853	Mixed receptive‐expressive language disorder, hyperactive, esotropia, known chromosome abnormality	Duplication	1–2
**M**	chrX:33158109–33273667	Obsessive compulsive disorder, dysmorphic craniofacial features, autism, attention‐deficit disorder	Duplication	1
**F**	chrX:26393764–35630680	Developmental disorders, developmental delay	Deletion	entire gene
**F**	**chrX:31764087–31864634**	**Tetralogy of Fallot, feeding difficulties, chronic lung disease, apnea**	**Deletion**	**49–51**
**M**	**chrX:31764087–31864634**	**Short stature, microcephaly, failure to thrive (** **Table** 3 **, family 10)**	**Deletion**	**49–51**
**M**	chrX:31961632–32034270	Short stature, hypotonia	Deletion	45
**F**	**chrX:31764087–31864634**	**Microcephaly, intrauterine growth restriction, failure to thrive**	**Deletion**	**49–51**
**F**	**chrX:31764087–31864634**	**Macrocephaly, hypotonia, hydrocephalus, dysmorphic craniofacial features, developmental delay**	**Deletion**	**49–51**
**M**	chrX:32604077–33507931	Language disorder	Duplication	1–13
**F**	**chrX:31764087–31864634**	**Short stature, obesity, learning disability**	**Deletion**	**49–51**
**M**	chrX:32041580–32556252	Developmental delay	Deletion	18–44
**M**	**chrX:31778941–31883357**	**Autism spectrum disorder (** **Table** 3 **, family 1)**	**Deletion**	**49–51**
**F**	**chrX:31764087–31864634**	**Optic nerve hypoplasia, developmental delay**	**Deletion**	**49–51**
**F**	**chrX:31764087–31864634**	**Muscle weakness**	**Deletion**	**49–51**
**M**	**chrX:31764087–31864634**	**Speech delay, esotropia, developmental delay (** **Table** 3 **, family 3)**	**Deletion**	**49–51**
**M**	chrX:31737146–31759752	Hypotonia, failure to thrive, developmental delay	Deletion	52
**F**	chrX:26684761–31848318	Autism spectrum disorder, developmental delay, sensory disorder, mixed receptive‐expressive language disorder	Duplication	50–79
**M**	chrX:31452021–31567623	Epilepsy, autism	Deletion	56–60
**F**	chrX:32703211–32785461	Mixed receptive‐expressive language disorder, lack of coordination, developmental delay	Deletion	8–9
**M**	**chrX:31764087–31864634**	**Hypotonia, Chiari malformation, attention‐deficit hyperactivity disorder (** **Table** 3 **, family 11)**	**Deletion**	**49–51**
**M**	chrX:31315530–31647841	Advanced maternal age	Duplication	55–62
**F**	chrX:30979608–31366745 chrX:32703211–32785461	Mixed receptive‐expressive language disorder, lack of coordination, developmental delay	Duplication	8–9 and 61–79

*Note*: Rows in bold reflect patients with the *DMD* exon 49–51 deletion.

In most cases, genetic testing, most commonly CMA, had initially been ordered as part of evaluations for developmental delays, autism, or other congenital findings (i.e. congenital heart defect, short stature). Three patients had diagnoses of autism, and a fourth had a diagnosis of Asperger's syndrome. One patient's genetic testing (from family 15) had been ordered subsequent to identification of elevated CK levels.

One patient had undergone a muscle biopsy, and Western blot performed on muscle tissue showed 20–100% expression of dystrophin of decreased molecular size. The highest measured CK level was 1477 (reference level 22–210 unit/L), and most patients had mildly elevated or normal CK levels. In our Genetics and Genomics Diagnostic Laboratory, this deletion accounted for 19% (4/21) cases of dystrophinopathies diagnosed in males by CMA from 1/1/2010–12/31/2020 (Table 4).

## 5 DISCUSSION

This case describes an unexpected outcome of maternal carrier testing for a familial *DMD* variant: detection of a second *DMD* variant previously unknown in the family. This represents the fifth published case to our knowledge of a female with multiple *DMD* variants confirmed in *trans*.

While the presence of multiple *DMD* variants in a family has been reported previously (Morandi et al., 1995), maternal transmission of two different *DMD* variants has not been previously described. In order to reconcile the apparently mild phenotype of our patient with her genotype of biallelic *DMD* variants, we performed a literature review to ascertain publications that describe the exon 49–51 deletion, as well as a retrospective chart review of individuals with this deletion from our institution.

While BMD was the most frequent diagnosis observed during our literature review, the phenotype resulting from a hemizygous *DMD* deletion of exons 49–51 is variable, with at least one asymptomatic case and at least six cases with DMD. Males in our retrospective chart review appeared to have a generally milder phenotype than those identified during the literature review. However, patients in our chart review were all under age 16 years at last evaluation, so our cohort did not contain data for adult patients. Interestingly, most unrelated male patients in our chart review were initially diagnosed by CMA rather than through DMD testing ordered based on clinical suspicion of dystrophinopathy. In fact, this particular deletion was a frequent finding compared to other deletions or duplications in the *DMD* gene found in the laboratory by CMA; of 32 exonic *DMD* copy number changes detected by CMA over the past 10 years in unrelated males or females, the exon 49–51 deletion comprised 10 (31%) of cases (Table 4).

Since CMA is typically ordered as a first‐line test to assess for developmental delays and is not specifically indicated as a first‐line test for dystrophinopathies, this suggests that clinical suspicion for a dystrophinopathy at the outset of testing for these patients was low. Considering the few reports of this deletion in the published literature, the apparent frequency at which this deletion is identified in our laboratory by CMA suggests that some cases of this deletion may go unrecognized, perhaps due to mild or absent clinical features. It is difficult to conclude whether the developmental delays noted in some of our patients could be attributed to the underlying *DMD* deletion or whether they could have a separate cause.

Anecdotally, we have encountered this *DMD* deletion in females in the setting of expanded carrier screening, although we did not locate any studies addressing *DMD* carrier results in this setting that might allow us to quantify its frequency. Large‐scale testing has been performed in at least one study that recruited women with hyperCKemia for *DMD* testing to assess carrier status. In that study, six dystrophinopathy carriers were identified at a carrier rate of 1:4088, but the identified *DMD* variants did not include the exon 49–51 deletion (Han et al., 2020).

A separate study from a large commercial laboratory summarized copy number variations (CNVs) identified during clinical testing of 384 genes. This summary included all diagnostic tests, and was not limited to carrier screening. *DMD* deletions or duplications were identified in 30 patients (24 females, six males). The *DMD* exon 49–51 deletion accounted for seven of the 30 patients (23%) (six females, one male). While individual clinical information was not included, this was the most frequent *DMD* CNV identified in the study (Truty et al., 2019).

In our institutional chart review, four of our 15 patients (26%) had diagnoses of autism or Aspberger's syndrome. Neurobehavioral diagnoses, including autism spectrum disorder, are known to occur more frequently in the setting of dystrophinopathies compared to the general population, and are diagnosed in up to 20% of patients (Banihani et al., 2015; Fujino et al., 2018; Hendriksen & Vles, 2008). While the proportion of our patients with autism spectrum disorders is higher than has been observed in previous studies, our small sample size and the lack of further autism‐specific genetic testing in all of the patients in our cohort makes it difficult to discern the relative contribution of the *DMD* exon 49–51 deletion to autism risk.

For our patient with biallelic exon 49–51 and exon 45–47 deletions, we were limited in our ability to functionally characterize dystrophin expression without skeletal muscle tissue, so it is not possible to account for her phenotype through assessment of dystrophin expression. However, literature and chart reviews yielded a particularly variable phenotype resulting from the exon 49–51 deletion. In some cases it appears to result in mild or absence of symptoms in males, suggesting production of some functional dystrophin protein, as would be expected for an in‐frame deletion. One patient from our chart review had Western blot studies performed on skeletal muscle tissue, which showed 20–100% dystrophin expression with decreased molecular size. The production of some functional dystrophin protein may offer an explanation for mild symptoms in a female who additionally carries a second in‐frame *DMD* variant on the opposite allele.

## 6 CONCLUSIONS

This case describes a female patient with biallelic *DMD* deletions who has three sons with Becker muscular dystrophy due to two different *DMD* deletions. While one of the two deletions (exons 45–47) is well‐described in the published literature in association with BMD, we performed a retrospective chart review and literature review of the second deletion (exons 49–51) in order to better understand the resulting phenotypic spectrum. Notably, a variety of molecular methods were utilized across the studies collected during our literature review, and it is not possible to exclude inaccuracies in genotyping that may have resulted from older methodologies. However, this review demonstrated a variable but generally mild phenotype ranging from DMD to absence of symptoms, which may contribute to our understanding of the mild phenotype found for our female patient. Additionally, this variant is commonly identified by CMA rather than by *DMD*‐specific testing, suggesting a dystrophinopathy is not initially specifically suspected at the time of testing.

This case raised two unique genetic counseling issues: (1) an unexpected second variant was identified during familial carrier testing, and (2) familial testing resulted in a new diagnosis in a family member who was previously thought to be unaffected. While targeted analysis for the known familial exon 45–47 deletion was requested for this case, the proximity of the two deletions to each other resulted in visualization of the second deletion during analysis. While the personal and family history of the patient was not suggestive of a second *DMD* variant, and the presence of a single variant in a family does not increase the likelihood of a second variant, it is reasonable to include a discussion about the possibility of unexpected results during pre‐test counseling for carrier testing for dystrophinopathies, given their prevalence in the general population. The possibility of unexpected results during maternal carrier testing would include the possibility of unexpected diagnoses in additional family members, such as our patient's middle son, who previously was perceived by the family to be unaffected by comparison to his brothers.

## AUTHOR CONTRIBUTIONS

Elizabeth A. Ulm designed the study, Elizabeth A. Ulm and Chinmayee B. Nagaraj participated in data collection and review, all authors participated in interpretation of the data, manuscript drafts, and criticalrevisions.

## CONFLICT OF INTEREST

The authors have no conflicts of interest to declare.

## ETHICS STATEMENT

This chart review was reviewed by the Cincinnati Children's Hospital Medical Center Institutional Review Board and was determined to be exempt (8/19/2020) (IRB ID: 2020–0656).

Exempt Category (4) Secondary research on data or specimens (no consent required).

Waiver of HIPAA: The IRB has granted a waiver from the requirement to obtain an authorization for the use and/or disclosure of protected health information (PHI).

## Data Availability

Data sharing is not applicable to this article as no new data were created or analyzed in this study.
